# Recurrence in acromegaly: two tertiary centers experience and review of the literature

**DOI:** 10.1007/s40618-024-02321-6

**Published:** 2024-03-19

**Authors:** A. Cremaschi, E. Sala, E. Lavezzi, G. Carosi, G. Del Sindaco, A. Mangone, R. Mungari, A. Pagnano, R. Indirli, E. Ferrante, G. Mazziotti, M. Locatelli, G. Lasio, M. Arosio, A. G. Lania, G. Mantovani

**Affiliations:** 1 Endocrinology Unit, Fondazione IRCCS Ca’ Granda Ospedale Maggiore Policlinico, Milan, Italy; 2 Department of Medical Sciences and Community Health, University of Milan, Milan, Italy; 3 Endocrinology and Diabetology Unit, IRCCS Humanitas Research Hospital, Rozzano, Italy; 4 Department of Biomedical Sciences, Humanitas University, Pieve Emanuele, Italy; 5 Neurosurgery Unit, Fondazione IRCCS Ca’ Granda Ospedale Maggiore Policlinico, Milan, Italy; 6 Department of Pathophysiology and Transplantation, University of Milan, Milan, Italy; 7 Neurosurgery Unit, IRCCS Humanitas Research Hospital, Rozzano, Italy

**Keywords:** Acromegaly recurrence, Acromegaly review, Pitituary adenoma, GH adenoma

## Abstract

**Background:**

Recurrence of acromegaly after successful surgery is a rare event, but no clear data are reported in the literature about its recurrence rates. This study aimed to evaluate the recurrence rate in a series of acromegalic patients treated by transsphenoidal surgery (TSS) with a long follow-up.

**Methods:**

We retrospectively analyzed data from 283 acromegalic patients who underwent TSS at two pituitary units in Milan (Fondazione IRCCS Ca’ Granda Ospedale Maggiore Policlinico and IRCCS Humanitas Research Hospital). The diagnosis and recurrence of acromegaly were defined by both elevated IGF-1 levels and a lack of GH suppression based on appropriate criteria for the assay used at the time of diagnosis.

**Results:**

After surgery, 143 patients (50%) were defined as not cured, 132 (47%) as cured and 8 (3%) as partially cured because of normalization of only one parameter, either IGF1 or GH. In the cured group, at the last follow-up (median time 86.8 months after surgery), only 1 patient (0.7%) showed full recurrence (IGF-1 + 5.61 SDS, GH nadir 1.27 µg/l), while 4 patients (3%) showed only increased IGF1. In the partially cured group at the last follow-up, 2/8 (25%) patients showed active acromegaly (IGF-1 SDS + 2.75 and + 3.62; GH nadir 0.6 and 0.5 µg/l, respectively).

**Conclusions:**

In the literature, recurrence rates range widely, from 0 to 18%. In our series, recurrence occurred in 3.7% of patients, and in fewer than 1%, recurrence occurred with elevation of both IGF-1 and the GH nadir. More frequently (25%), recurrence came in the form of incomplete normalization of either IGF-1 or GH after surgery.

## Introduction

Acromegaly is a chronic and rare disease characterized by GH hypersecretion, most frequently caused by GH-secreting pituitary tumors arising from somatotroph cells [1]. Rare causes of acromegaly are pituitary carcinoma and ectopic production of GHRH by both central hypothalamic tumors and peripheral neuroendocrine tumors [2]. Active acromegaly causes a twofold increase in mortality, and malignancies are the leading cause of death, followed by cardiovascular disease [3, 4]. Biochemical control of the disease has been confirmed to reduce the excess mortality in the general population [2, 4].

The Endocrine Society guidelines recommend transsphenoidal (TSS) surgery as first-line therapy in most patients [1]. The outcomes of the procedure are dependent on various factors, such as the tumor size, degree of invasion and experience of the surgical team [5]; with experienced pituitary surgeons, TSS surgery results in an initial remission rate ranging from 40 to 85%, depending on the series and the size of the tumor [1].

The criteria to define both diagnosis and remission of acromegaly have changed over the years, mainly due to the availability of more sensitive GH assays [6]. As for remission, the current criteria were established by the Consensus Conference in 2010 and confirmed by the 2014 Clinical Practice Guidelines as IGF-1 levels in the age- and sex-adjusted normal range and, in patients with elevated or equivocal serum IGF-1 levels, random GH levels < 1.0 mcg/l using commercially available immunoassays 12 weeks after surgery [1, 7]. When random GH levels are detectable, current guidelines suggest measuring the GH nadir after a glucose load (OGTT), with levels greater than 0.4 mcg/liter in an ultrasensitive chemiluminescent assay (CLIA) defining active disease [1, 7].

In the past, when GH levels were measured with classical radioimmunoassay (RIA) [8], random GH levels < 2.5 mcg/l and nadir GH levels after a glucose load < 2 mcg/L were accepted as standards for the remission of disease [6]. In 1999, the Consensus Conference in Cortina defined cure criteria as IGF-1 levels in the age- and sex-adjusted normal range and a random GH value < 2.5 mcg/l or a GH nadir after OGTT < 1 mcg/l when immunoradiometric assays (IRMA) became available [8–10]. These past assays were competitive and were relatively insensitive, typically exhibiting a low detection limit [8].

It is critical to differentiate patients with persistent disease from those in remission to start with the correct therapy and follow-up. To do so, the timing of biochemical testing after surgery is very important because IGF-1 has a longer half-life and therefore takes more time to decrease and normalize than GH does [5].

Recurrence is defined as a relapse into GH hypersecretion after initial postsurgical remission, as defined by the criteria for cure [11]. In the literature, the reported recurrence rates are variable, and studies reporting recurrence rates are heterogeneous in terms of population, criteria for the definition of cure, and timing of biochemical testing after surgery. Many of these papers report surgical series in which recurrence was not the main aim of the study (Table 1).

**Table 1:** Literature review of acromegaly recurrence

	Population	Time period and nature of study	Surgical Series	Recurrence Rate (%)	Mean/median follow-up time	Criteria for remission	Time of first evaluation after surgery	Time of recurrence	Risk factors for recurrence
Nomikos et al. [13]	668	1982–2001 retrospective	Yes	0,4	Mean 126 ± 74 months	GHr < 2,5 μg/L + GHn < 1 μg/L + IGF1	7 days; 3 months	3 and 6 years	Remission criteria used
Freda et al. [14]	97	1996–2020 Prospective	No	14	Mean 9,34 ± 6.4 years	GHn < 0,4 μg/L + IGF1	3 months	Median of 4.5 year (range 2,8–24 year)	Abnormal GH levels at OGTT (GH 0,14–0,4)
Cunha et al. [5]	54	2001–2019 retrospective	Yes	0 in cured 13 (8,3 BDI; 33,3 in BDII^*^) discrepancies	Median of 39 (range 12–126) months	GHr < 1 μg/L + IGF1 (Discrepancy if only 1 criteria)	3 months	NA	IGF1 < 1,25 ULN °
Biermasz et al. [15]	59	1977–1988 retrospective	Yes	18,5	Mean 16 ± 0.8 years	IGF1 + mean GH day-profile or GHr < 2,5 μg/L + GHn	7–10 days; 6 months	6 months – 3 / 4 years – 10 years	Preoperative mean GH concentration
Beauregard et al. [16]	103	1970–1999 retrospective	No	6,8	Mean 13,4 (range 10–29) years	GHr < 2,5 μg/L ± GHn < 1 μg/L ± IGF1	NA	Mean of 4 years	–
Krieger et al. [17]	181	1973–1990 retrospective	Yes	0,8	Mean 5 years	GHr < 2 μg/L	1 day	NA	–
Ronchi et al. [6]	40	2000–2004 retrospective	No	2	Mean 14,3 ± 4,2 years	IGF1 + GHr < 2,5 ± TRH and GnRH test	Median 3 months (range 3–9 months)	NA	–
Banerji et al. [18]	101	1992–2005 prospective	Yes	7	Median 84 (range 3–132) months	GHr < 2,5 μg/L + GHn < 1 μg/L No IGF1	6 weeks	NA	Tumor size
Roelfsema et al. [19]	3548	Review	Mixed	4,5 0,007 pts/yrs	Mean 6,36 ± 0,58 years	Mixed	Mixed	1–10 years	Postoperative GHr and GHn; Paradoxical GH increase after TRH infusion
Liang Lv et al [20]	94	2008–2017 retrospective	Yes	10	Median 35 (range 13–129) months	GHn < 0,4 μg/L + IGF1	3 months	NA	Postoperative GHn levels (1 week after surgery)
Shen et al. [21]	133	2013–2014 retrospective	Yes	6,5	Median 48 (range 12–84) months	GHn < 1 μg/L + IGF1	3 months	12, 36 and 54 months	tumor proliferation (Ki-67); GHn at surgical remission
Kristof et al. [22]	102	retrospective	Yes	2,08	Mean 5,06 years (range 3 months-16.9 years)	GHn < 1 μg/L + IGF1	3 months	4,6 and 4,9 years	–
De et al. [23]	90	1980–2001 retrospective	No	0	Mean 10,9 years (range 6 months – 20 years)	GHr < 2,5 μg/L + GHn < 1 μg/L + IGF1	4–6 weeks	–	–
Rotermund et al. [24]	280	2008–2015 retrospective	Yes	12,2	Median 29 (range 1–120) months	IGF1 + GHr < 1 μg/L or GHn < 1 μg/L	1 and 3 days	NA	–
Sun et al. [25]	86	2006–2011 retrospective	Yes	25	Mean 13,4 ± 15,8 months	GHr < 1 μg/L or GHn < 0,4 μg/L	NA	38 and 22 months	SSTR2A expression
Shimon et al. [26]	98	1990–1999 retrospective	No	8	Mean 3,9 ± 2,5 years	GHn < 2 μg/L + IGF1	NA	1 year	–
Babu et al. [27]	58	2005–2013 retrospective	Yes	2,3	Mean 64 ± 32,2 months	GHn < 1 μg/L + IGF1	6–12 weeks	< 12 months	–
Fernandez Mateos et al. [28]	548	1975–2015 retrospective	Yes	6,5^#^	Mean 3,3 ± 2,3 years	GHr < 2 μg/L + GHn < 1 μg/L + IGF1	3–6 months	4, 7, 8 and 12 years	–
Diri et al. [29]	108	Retrospective	No	14,9	Mean 44,8 (range 24–59) months	GHn < 1 μg/L + IGF1	3 months	NA	Initial tumor size; compression of optic chiasm; diagnosis IGF1 levels
Kreutzer et al. [30]	57	1992–1998 retrospective	Yes	2,4	Mean 37,7 (range 12–88) months	GHr < 2,5 μg/L + GHn < 1 μg/L + IGF1	6 weeks (range 1–12 months)	NA	–
Shirvani et al. [31]	130	1992–2010 retrospective	Yes	10,8	Mean 35,9 (range 6–169) months	GHr < 2,5 μg/L + IGF1	1 day	Mean 31,4 (range 10–100) months	–

*IGF1* levels in age- and sex-adjusted normal range, *GHr* GH random levels, *GHn* nadir GH levels after OGTT

^*^ BDI: Biochemical discrepancy type I (normal IGF1 levels + random serum GH ≥ 1.0 μg/L); BDII: Biochemical discrepancy type II (high IGF-I + random serum GH < 1.0 μg/L)

° IGF-I levels up to 1,25-fold the ULN determined 3 months after TNS in acromegaly patients was associated with long-term remission, regardless of GH levels, even in patients initially considered as surgical failure. IGF-I ≤ 1,25-fold the ULN had 100% sensitivity and 96% specificity to predict long-term remission

^#^ Recurrence rate was calculated on 61 patients initially in remission with available data 15 years after surgery (4/61)

To date, there are no clear clinical suggestions about the timing of long-term follow-up or the criteria for discontinuation. In our tertiary centers in Milan, most acromegalic patients, even when cured by surgery, stay under lifelong follow-up, which brings personal and social costs.

This study aimed to evaluate acromegaly patients successfully treated by TSS with a long follow-up history to establish the recurrence rate and the need for further follow-up.

## Methods and study design

We retrospectively analyzed the data of 283 consecutive acromegalic patients (168 F, mean age 44.2 ± 12.9) who underwent TSS for a GH-secreting pituitary tumor between 1980 and 2020 in two specialized centers in the city of Milan: Fondazione IRCCS Ca’ Granda Ospedale Maggiore Policlinico (245 patients) and IRCCS Humanitas Research Hospital (38 patients), who underwent at least one year of follow-up after surgery.

Hormonal data included IGF-1 and GH Nadir. The GH nadir was defined as the lowest GH value at any time during a 2-h oral OGTT. For this, blood samples were collected at 30, 60, 90, and 120 min after glucose load.

Study eligibility criteria included age greater than 18 years, a diagnosis of acromegaly based on the combination of both an elevated serum IGF-1 level and lack of GH suppression based on criteria appropriate for the assay used at the time of diagnosis: GH > 2 µg/L by RIA, GH > 1 µg/L by modern IRMA or GH > 0.4 µg/L by CLIA. They also had to have MRI evidence of pituitary adenoma and to have undergone transsphenoidal endonasal surgery (TSS).

In particular, in both units between 1980 and 1992, the RIA method was used (detection limit 0.3 ng/ml). Then, from 1992 to 2007, the IFMA method was used (AutoDelfia Kit; detection limit 0.01 ng/ml, 80/505). From 2007 to the present, we have used a CLIA, the Immulite 2000 or an Immulite assay comparable to the Immulite 2000 (detection limit 0.01 ng/ml; 2007–2010 standard 80/505, 2010-present standard 98/574). The standards used for calibration were IS 80/505 from 2008 to July 2010 and IS 98/574 from August 2010.

IGF-1 values are expressed as standard deviation scores (SDSs). We obtained SDS values according to the methods provided by Chanson and colleagues [12].

The local ethics committee (Comitato Etico Milano Area 2) approved this protocol study.

### Study design

All patients had a biochemical diagnosis of acromegaly according to the criteria used at the time of evaluation.

All patients underwent thinly sliced magnetic resonance imaging (MRI) or computerized tomography (CT) of the sellar region to evaluate the presence of a pituitary tumor. Tumors were classified by their maximum diameter as microadenomas (≤ 10 mm) or macroadenomas (> 10 mm).

All patients underwent TSS at one of the two neurosurgery units after the confirmation of the acromegaly diagnosis. 34 patients received medical treatment before surgery, in particular 19 (56%) with somatostatin analogues, 12 (35%) with dopamine agonists and 3 patients (9%) with pegvisomant.

We analyzed the data of all patients at the first follow-up after surgery, at least 12 weeks after the procedure. We defined patient outcomes as follows:

- cured when both normal serum IGF-1 levels and normal GH suppression after OGTT (defined by the criteria for the assay used at the time) were observed;

- partially cured when only IGF1 level or GH suppression was normalized;

-not cured in the case of both elevation of serum IGF-1 level and lack of GH suppression (defined by the criteria for the assay used at the time).

The cured and partially cured patients were evaluated at least annually for a median follow-up time of 86.8 months (IQR 31–172.9 months). Their status at the last available follow-up was analyzed here. Recurrence was defined by the presence of elevated IGF-1 levels in addition to a lack of GH suppression (defined by the criteria for the assay used at the time), and the occurrence data were analyzed at the time of recurrence and at the last available follow-up.

### Statistical analysis

Quantitative variables are expressed as mean ± SD when normally distributed, while quantitative variables that were not normally distributed are expressed as median (interquartile range (IQR)). Qualitative variables are expressed as number and percentages (%).

Comparisons between two groups of data were analyzed by the paired or unpaired t-tests, when variables were normally distributed. Comparisons between categorical variables were assessed by the chi-square test, or by Fisher’s exact test when the expected values were < 5. Data were analyzed using GraphPad Prism (version 5.0, La Jolla, CA, USA) or SPSS (PASW Version 19.0, SPSS Inc., Chicago, IL, USA). P values (*p*) < 0.05 were considered statistically significant.

## Results

All 283 patients had preoperative confirmation of acromegaly (mean IGF1: + 13.7 ± 9.2 SDS, median GH nadir 7.6 IQR 2.8–17.9 µg/L). MRI confirmed the presence of a pituitary tumor in all patients (192 macro/91 micro), all patients underwent TSS, and all patients had pathological confirmation of a pituitary tumor positive for GH on immunohistochemical staining.

### First follow-up after surgery

At the first follow-up after surgery (median distance of 3 months IQR 2–3 months), we defined 143 patients (50%) as not cured, 132 patients (47%) as cured, and 8 patients (3%) as partially cured, of whom 6 patients had normal IGF1 values but no GH suppression after OGTT and 2 patients had elevated IGF1 levels and normal GH suppression**.**

### Cured patients at last follow-up

The median follow-up available for the total population of the study was 104.9 months (IQR 45.6–212.1 months). Within the group of cured patients, at the last available follow-up (median follow-up time 86.8 months IQR 31–172.9 months after surgery), 5 out of 132 (3.7%) showed abnormalities of hormone levels.

In particular, 1 patient (0.7%) showed a biochemical status of active acromegaly with both elevated IGF1 levels (IGF1 + 5.61 SDS) and lack of GH suppression after OGTT (GH nadir 1.27 µg/L) 15 months after surgery. Both evaluations of IGF1 and GH levels, after surgery and at the time of recurrence, were analyzed in the same laboratory with the same assay (CLIA).

Four other patients (3%) showed an increase in IGF1 levels during follow-up despite consistently normal GH suppression. After multiple confirmations of isolated IGF1 levels above the upper normal limit (mean IGF1 SDS + 3.65), with a mean follow-up of 81 months from surgery, these patients started medical therapy for acromegaly.

All patients with biochemical partial or complete recurrence underwent a new MRI of the pituitary region. In all cases, this demonstrated neuroradiological stability with no signs of tumor recurrence or regrowth (Table 2). At the time of diagnosis and before surgery, the only patient with true recurrence had a microadenoma, while of the other 4 patients 2 had a microadenoma (50%) and 2 had a macroadenoma (50%).

**Table 2:** Individual hormonal and radiological data of patients, both cured and partially cured at first follow-up, who showed recurrence of disease after surgery

	Sex	Age at diagnosis	After TNS surgery	At time of recurrence	MRI imaging at follow-up	Time of recurrence (months after TNS)
Patients cured at first follow-up after surgery						
1	M	59	IGF1 -0.15 SDS GHn 0.09 µg/L	IGF1 5.61 SDS GHn 1.27 µg/L	Stable residual tumor	15
2	F	23	IGF1 0.93 SDS GHn 0.06 µg/L	IGF1 5.07 SDS GHn 0.18 µg/L	Empty sella	157.8
3	M	50	IGF1 1.12 SDS GHn 0.20 µg/L	IGF1 3.85 SDS GHn 0.16 µg/L	Stable residual tumor	134
4	M	41	IGF1 0.26 SDS GHr 0.8 µg/L	IGF1 4.72 SDS GHn 0.50 µg/L	Stable residual tumor	28
5	F	36	IGF1 -0.40 SDS GHn 0.11 µg/L	IGF1 3.25 SDS NA	NA	5
Patients partially cured at first follow-up after surgery						
6	F	47	IGF1 2.01 SDS GHn 0.47 µg/L	IGF1 2.75 SDS GHn 0.60 µg/L	Empty sella	12
7	M	74	IGF1 0.76 SDS GHn 2.20 µg/L	IGF1 3.62 SDS GHn 0.50 µg/L	Stable residual tumor	37

### Partially cured at last follow-up

In the partially cured group, 2 (25%) patients showed, 12 and 37 months after surgery, a biochemical status of active acromegaly (IGF1 SDS + 2.75 and + 3.62; GH nadir 0.6 and 0.5 µg/l, both assessed with CLIA; all data are summarized in Table 2). In this group of patients, no signs of tumor regrowth were found on pituitary MRI **(**Table 2**).** Both recurrent patients started medical therapy for acromegaly after evidence of both elevated IGF1 and GH nadir levels.

Interestingly, one of the patients who experienced recurrence had normal IGF1 levels but a lack of GH suppression at the first follow-up after surgery, while the second patient showed normal GH suppression but elevated IGF1 levels.

Both patients had a macroadenoma at the time of diagnosis before surgery.

The percentage of recurrent patients in the partially cured group was significantly higher than that in the cured group (p = 0.008). We did not find any other significant difference between the recurrent group and the nonrecurrent group, which is likely due to the small number of patients in the sample. We also analyzed the possible role of different neurosurgeons, however, the two centers did not significantly differ in recurrence rates: in the first center of 111 patents cured or partially cured after surgery 5 had a recurrence (4.5%); in the second of the 21 patients cured or partially cured after surgery 2 had a recurrence (9.5%); p = 0.3. Our data suggest that an incomplete cure after surgery (considering both GH or IGF1 levels) is associated with a higher likelihood of recurrence.

Moreover, we did not find any significant difference in biochemical status after surgery or pathological findings between patients with and without complete recurrence in the partially cured group, again probably because of the small number of patients (IGF1 after surgery + 1.6 ± 0.6 SDS vs. + 1.3 ± 0.8 SDS, GH random 3.4 ± 0.3 vs. 2.7 ± 0.7, Ki67% was less than 1% in all patients).

### Literature review

We searched the PubMed database using the keyword “acromegaly and recurrence” for articles published between 2000 and 2021. Only full-text articles were included. The literature review showed 21 papers with data on recurrence rates or whose outcome was recurrence of acromegaly after pituitary surgery in adult patients [5, 6, 13–31] (Table 1). Series that included patients treated with stereotactic surgery were excluded. Of the 21 studies, 14 (66.7%) were surgical series. The majority were retrospective studies, only two were prospective studies, and one was a literature review. Recurrence rates varied from 0% to 18.5%, and the time of recurrence ranged from 6 months to 10 years. All rates, remission criteria and time of first evaluation are summarized in Table 1.

## Discussion and conclusions

Recurrence rates in acromegalic patients successfully treated by surgery range widely in the literature. Different series were published with a percentage of recurrence that ranges from 0% [13] to 18% [15] (Table 1).

Many reasons could underlie these important differences: the series vary in terms of numbers and population characteristics, timing of follow-up and, most importantly, the criteria used to define active acromegaly (all data are shown in Table 1). Many of the series are surgical ones, whose aim was to evaluate the surgical remission rather than recurrence after surgery and thus frequently there is little precision or attention to hormonal data and recurrence features. Other possible reasons for such a wide range may be the tumor dimension, invasion, and aggressiveness [32] or different genetic causes of acromegaly. In addition, when defining the correct recurrence rate, the timing of follow-up is crucial, especially the timing of the first follow-up after surgery. Guidelines suggest that the first follow-up should be at least 12 weeks after surgery in order to correctly categorize the outcome of surgery [1]. This timing takes into consideration the fact that the decline in IGF-1 levels is more delayed compared with the decline in GH values, likely due to the differential half-life of IGF-binding proteins [33]. An earlier follow-up after surgery may not reflect the real biochemical status and may be confusing for both patients and practitioners.

Moreover, in some series, the definition of recurrence was based on either IGF1 levels alone, random GH or GH suppression after OGTT (with different cutoffs), or the simultaneous presence of more than one of these parameters. As expected, stricter criteria are linked to a lower prevalence of recurrence than criteria that do not consider both IGF1 and GH suppression. For example, the paper by Biermasz and colleagues [15], which reports the highest percentage of recurrence (18%), analyzed a small cohort of patients, of whom 75% had MRI evidence of macroadenomas and 15% had evidence of invasive tumors. The timing of the first follow-up after surgery was not specified; it was generically defined as early postoperative, and only one parameter, either GH or IGF1, defined recurrence. A small population with aggressive tumors, with no strict diagnostic criteria and a possible misleading biochemical definition after surgery, will likely be linked to a higher percentage of recurrence (data shown in Table 1) [15].

In contrast, we found less than 1% true recurrence (0.7%), in agreement with the largest series published to date on this topic, by Nomikos and colleagues, who reported a recurrence rate of 0.4% in a cohort of 506 acromegalic patients with strict diagnostic criteria applied three months after surgery [13].

These very low recurrence rates are also in agreement with other previous studies using similar methodological approaches in terms of the timing of follow-up and diagnostic criteria, thus confirming that acromegaly recurrence is very rare in patients successfully treated with surgery [5, 13, 17].

A particular situation in our cohort is represented by the 3% of patients who, after multiple confirmations of elevated IGF1 levels, started a medical therapy for their partial recurrence despite the persistence of normal GH suppression. During medical therapy, we could not repeat testing for GH suppression, so we are not able to exclude the possibility that these patients were evaluated during an early phase of relapse and that over time they would have shown complete biochemical recurrence. On this topic, a recent and interesting long-term prospective study by Freda and colleagues demonstrated that by performing repeatedly OGTT in a small series of acromegalic patients defined as cured after surgery, a few of them had an elevation in the GH nadir levels after OGTT over time (using a cutoff relative to the 97.5th percentile of nadir GH in 100 healthy subjects, which was ≤ 0.14 µg/l with DSL IRMA), to the point of even reaching a biochemical definition of complete recurrence (GH nadir > 0.4 µg/l). Interestingly, recurrence was more likely in patients who had a GH nadir > 0.14 µg/l on initial postsurgical testing [14]. This study further underlines the importance of performing OGTT in patients after surgery to correctly interpret the hormonal status and plan the follow-up. Unfortunately, the retrospective nature of our study could not better investigate these aspects.

Moreover, in our retrospective cohort, the small number of recurrences did not allow us to investigate, with statistically significant results, the presence of predictive factors. Our study reports an important recurrence rate (25%) in patients with incomplete biochemical remission after surgery, with a statistically significant difference compared to the group of patients completely cured after surgery (p = 0.008). Once more, as the number of patients with incomplete remission was small, we could not define whether values of GH or IGF1 were more important in the postoperative evaluation. Nevertheless, our data underline the need to focus on incompletely cured patients.

Overall, these findings, as also suggested by Freda and colleagues, support the role of a correct and complete postsurgical follow-up, suggesting the need to perform an OGTT in all patients, as opposed to guidelines that recommend performing OGTT only in patients with basal random GH levels > 1 µg/l [14].

GH measurement and its suppressibility by OGTT are not perfect measures, since current diagnostic GH nadir values should probably be better individualized according to age, sex, BMI and estrogenic status [34, 35]. This could be a factor that limited our ability to identify patients with altered GH secretion after surgery and at the time of recurrence.

Finally, our study suggests that recurrence, when present, is limited to the biochemical status. Interestingly, none of our patients showed either tumor regrowth or neuroradiological modification. This might have important practical clinical implications because, if confirmed by further larger studies, it could indicate the possibility of planning a biochemical follow-up alone without the need for radiological imaging, reducing the patient’s burden and costs.

In conclusion, our data confirm that acromegaly recurrence is very rare in patients successfully treated after surgery, even after a long follow-up time. More frequently (25%, p = 0.008), recurrence occurs in the form of incomplete normalization of either IGF1 or GH after surgery. As also suggested by previous studies using our approach, the right timing and the use of strict biochemical criteria at first follow-up after surgery are crucial to correctly define the surgical outcome and plan an appropriate follow-up. Indeed, our data suggest that most acromegalic patients with complete remission after surgery may be considered definitely cured, with no need for strict follow-up. In contrast, patients with partial remission after surgery need strict follow-up.

## Limitations

We acknowledge that this study is limited by the retrospective nature of the analysis. Moreover, our data were collected throughout a follow-up period of almost 40 years, during which time laboratory assays and biochemical criteria have changed several times reflecting technical improvements. Finally, the relatively small number of recurrences and partial remissions limits the strength of our conclusions and precludes a definitive identification of prognostic factors and clinical suggestions.

## References

[1] Katznelson L et al (2014) Acromegaly: an endocrine society clinical practice guideline. J Clin Endocrinol Metab 99(11):3933–3951. 10.1210/jc.2014-2700 25356808 10.1210/jc.2014-2700

[2] Melmed S (2006) Acromegaly. New Engl J Med 355:2558 17167139 10.1056/NEJMra062453

[3] Giustina A et al (2020) A consensus on the diagnosis and treatment of acromegaly comorbidities: an update. J Clin Endocrinol Metab 105(4):e937–e946. 10.1210/clinem/dgz096 10.1210/clinem/dgz096 31606735

[4] Arosio M et al (2012) Predictors of morbidity and mortality in acromegaly: an Italian survey. Eur J Endocrinol 167(2):189–198. 10.1530/EJE-12-0084 22596288 10.1530/EJE-12-0084

[5] da Cunha MLV, Borba LAB, Boguszewski CL (2020) Random Gh and Igf-I levels after transsphenoidal surgery for acromegaly: relation with long-term remission. Endocrine 68(1):182–191. 10.1007/s12020-020-02227-2 32078118 10.1007/s12020-020-02227-2

[6] Ronchi CL et al (2005) Long-term evaluation of postoperative acromegalic patients in remission with previous and newly proposed criteria. J Clin Endocrinol Metab 90(3):1377–1382. 10.1210/jc.2004-1974 15585548 10.1210/jc.2004-1974

[7] Giustina A et al (2010) A consensus on criteria for cure of acromegaly. J Clin Endocrinol Metab 95(7):3141–3148. 10.1210/jc.2009-2670 20410227 10.1210/jc.2009-2670

[8] Bidlingmaier M, Strasburger CJ (2007) Growth hormone assays: current methodologies and their limitations. Pituitary 10(2):115–119. 10.1007/s11102-007-0030-1 17429592 10.1007/s11102-007-0030-1

[9] Starnoni D, Daniel RT, Marino L, Pitteloud N, Levivier M, Messerer M (2016) Surgical treatment of acromegaly according to the 2010 remission criteria: systematic review and meta-analysis. Acta Neurochir (Wien) 158(11):2109–2121. 10.1007/s00701-016-2903-4 27586125 10.1007/s00701-016-2903-4

[10] Giustina A et al (2000) Criteria for cure of acromegaly: a consensus statement. J Clin Endocrinol Metab 85(2):526–529. 10.1210/jcem.85.2.6363 10690849 10.1210/jcem.85.2.6363

[11] Del Porto LA, Liubinas SV, Kaye AH (2011) Treatment of persistent and recurrent acromegaly. J Clin Neurosci 18(2):181–190. 10.1016/j.jocn.2010.10.003 21167718 10.1016/j.jocn.2010.10.003

[12] Chanson P et al (2016) Reference values for IGF-I serum concentrations: comparison of six immunoassays. J Clin Endocrinol Metab 101(9):3450–3458. 10.1210/jc.2016-1257 27167056 10.1210/jc.2016-1257 PMC5054194

[13] Nomikos P, Buchfelder M, Fahlbusch R (2005) The outcome of surgery in 668 patients with acromegaly using current criteria of biochemical ‘cure.’ Eur J Endocrinol 152(3):379–387. 10.1530/eje.1.01863 15757854 10.1530/eje.1.01863

[14] Freda PU et al (2021) Prognostic value of nadir GH levels for long-term biochemical remission or recurrence in surgically treated acromegaly. Pituitary 24(2):170–183. 10.1007/s11102-020-01094-4 33124000 10.1007/s11102-020-01094-4 PMC7969360

[15] Biermasz NR, Dulken HV, Roelfsema F (2000) “Ten-Year Follow-Up Results of Transsphenoidal microsurgery in acromegaly. J Clin Endocrinol Metab 85(12):7 10.1210/jcem.85.12.7042 11134114

[16] Beauregard C, Truong U, Hardy J, Serri O (2003) Long-term outcome and mortality after transsphenoidal adenomectomy for acromegaly: *Transsphenoidal adenomectomy outcome and mortality for acromegaly*. Clin Endocrinol (Oxf) 58(1):86–91. 10.1046/j.1365-2265.2003.01679.x 12519417 10.1046/j.1365-2265.2003.01679.x

[17] Krieger MD, Couldwell WT, Weiss MH (2003) Assessment of long-term remission of acromegaly following surgery. J Neurosurg 98(4):719–724. 10.3171/jns.2003.98.4.0719 12691394 10.3171/jns.2003.98.4.0719

[18] Banerji D, Das N, Sharma S, Jindal Y, Jain V, Behari S (2016) Surgical management of acromegaly: long term functional outcome analysis and assessment of recurrent/residual disease. Asian J Neurosurg 11(3):261. 10.4103/1793-5482.145354 27366253 10.4103/1793-5482.145354 PMC4849295

[19] Roelfsema F, Biermasz NR, Pereira AM (2012) Clinical factors involved in the recurrence of pituitary adenomas after surgical remission: a structured review and meta-analysis. Pituitary 15(1):71–83. 10.1007/s11102-011-0347-7 21918830 10.1007/s11102-011-0347-7 PMC3296023

[20] Lv L et al (2019) Mammosomatotroph and mixed somatotroph-lactotroph adenoma in acromegaly: a retrospective study with long-term follow-up. Endocrine 66(2):310–318. 10.1007/s12020-019-02029-1 31368083 10.1007/s12020-019-02029-1

[21] Shen M et al (2021) 2010 versus the 2000 consensus criteria in patients with normalised insulin-like growth factor 1 after transsphenoidal surgery has high predictive values for long-term recurrence-free survival in acromegaly”. J Neuroendocrinol. 10.1111/jne.12958 33998086 10.1111/jne.12958

[22] Kristof RA, Grote A, Redel L, Neuloh G, Klingmüller D, Schramm J (2011) The common consensus criteria have high predictive values for long-term postoperative acromegaly remission. Acta Neurochir (Wien) 153(1):19–25. 10.1007/s00701-010-0790-7 20845050 10.1007/s00701-010-0790-7

[23] De P et al (2003) Transsphenoidal surgery for acromegaly in wales: results based on stringent criteria of remission. J Clin Endocrinol Metab 88(8):3567–3572. 10.1210/jc.2002-021822 12915637 10.1210/jc.2002-021822

[24] Rotermund R et al (2020) Real-life analysis of 280 patients with surgically treated acromegaly: a single-center experience from 2008 to 2015. Neurosurg Focus 48(6):E9. 10.3171/2020.3.FOCUS2061 32480363 10.3171/2020.3.FOCUS2061

[25] Sun H, Brzana J, Yedinak C, Gultekin S, Delashaw J, Fleseriu M (2013) Factors associated with biochemical remission after microscopic transsphenoidal surgery for acromegaly. J Neurol Surg Part B Skull Base 75(01):047–052. 10.1055/s-0033-1354578 10.1055/s-0033-1354578 PMC3912146 24498589

[26] Shimon I, Ram Z (2001) Transsphenoidal surgery for acromegaly: endocrinological follow-up of 98 patients. Neurosurgery 48(6):1239–1245. 10.1097/00006123-200106000-00008 11383725 10.1097/00006123-200106000-00008

[27] Babu H et al (2017) Long-term endocrine outcomes following endoscopic endonasal transsphenoidal surgery for acromegaly and associated prognostic factors. Neurosurgery 81(2):357–366. 10.1093/neuros/nyx020 28368500 10.1093/neuros/nyx020

[28] Fernández Mateos C, García-Uria M, Morante TL, García-Uría J (2017) Acromegaly: surgical results in 548 patients. Pituitary 20(5):522–528. 10.1007/s11102-017-0813-y 28589294 10.1007/s11102-017-0813-y

[29] Diri H, Ozaslan E, Kurtsoy A, Bayram F (2020) A single-center observational study assessing the predictive factors associated with the prognosis of acromegaly. Growth Horm IGF Res 55:101342. 10.1016/j.ghir.2020.101342 32916586 10.1016/j.ghir.2020.101342

[30] Kreutzer J, Vance ML, Lopes MBS, Laws ER (2001) Surgical management of GH-secreting pituitary adenomas: an outcome study using modern remission criteria. J Clin Endocrinol Metab 86(9):4072–4077. 10.1210/jcem.86.9.7819 11549628 10.1210/jcem.86.9.7819

[31] Shirvani M, Motiei-Langroudi R (2014) *Transsphenoidal* surgery for growth hormone-secreting pituitary adenomas in 130 patients. World Neurosurg 81(1):125–130. 10.1016/j.wneu.2013.01.021 23313263 10.1016/j.wneu.2013.01.021

[32] Zhang S et al (2022) Predictors of postoperative biochemical remission in lower Knosp grade growth hormone-secreting pituitary adenomas: a large single center study. J Endocrinol Invest 46(3):465–476. 10.1007/s40618-022-01873-9 36125731 10.1007/s40618-022-01873-9

[33] Freda PU (2003) Current concepts in the biochemical assessment of the patient with acromegaly. Growth Horm IGF Res 13(4):171–184. 10.1016/S1096-6374(03)00029-7 12914750 10.1016/S1096-6374(03)00029-7

[34] Arafat AM et al (2008) Growth hormone response during oral glucose tolerance test: the impact of assay method on the estimation of reference values in patients with acromegaly and in healthy controls, and the role of gender, age, and body mass index. J Clin Endocrinol Metab 93(4):1254–1262. 10.1210/jc.2007-2084 18171702 10.1210/jc.2007-2084

[35] Schilbach K et al (2019) Determinants of the growth hormone nadir during oral glucose tolerance test in adults. Eur J Endocrinol 181(1):55–67. 10.1530/EJE-19-0139 31096183 10.1530/EJE-19-0139

